# Life before birth: are the dice tossed for the rest of our lives?

**DOI:** 10.1007/s00787-012-0264-y

**Published:** 2012-03-02

**Authors:** Nanda N. J. Rommelse

**Affiliations:** 1 Karakter, Child and Adolescent Psychiatry University Center, Nijmegen, The Netherlands; 2 Department of Psychiatry, Radboud University Nijmegen Medical Centre, Nijmegen, The Netherlands; 3 Donders Institute for Brain, Cognition and Behaviour, Centre for Cognitive Neuroimaging, Radboud University Nijmegen, Nijmegen, The Netherlands

Without any exception, every human being has spent at least a considerable amount of time in the womb and has gone through the stressful process of being born. However, the quantity and quality of our life before birth varies tremendously between people. That is, some individuals survive birth after only 22 weeks of pregnancy [1] and others have the luxury of being born after spending more than 40 weeks in utero. Perhaps more importantly than the duration are the conditions of our stay: Do we receive enough and healthy nutrition? Are we exposed to toxics like tobacco, alcohol or drugs? Is the oxygen supply sufficient? Are we being subjected to high levels of stress hormones? Are we getting infected by bacteria or viruses? And then…birth. By some considered as the most stressful life event we will ever have to endure. A certain amount of stress is actually necessary for survival: The high level of hormones released during birth, which are also involved in the stress response, are believed to make the newborn more alert promoting the bonding process and, by extension, the child’s physical survival [2]. However, while a certain amount of stress is necessary for survival, extreme and/or prolonged stress can affect health adversely [3]. For example, it has been shown that people who had underwent an extremely stressful birth (defined as vaginal delivery with fetal suffering or forceps use, urgent cesarean section indicated by fetal suffering or twin birth with death of the other fetus) were 5 times more likely to have developed obsessive–compulsive disorder (OCD) in the year following a stressful life event compared to OCD patients without an extremely stressful birth [4]. In other words, the former group had more often a stress-event-related onset of OCD compared to the latter group, indexing the possibility that a very stressful birth creates a life-long enhanced vulnerability to stress and an enhanced risk of developing anxiety related symptoms, such as OCD. Animal studies also suggest that a single aversive life event may be capable of permanently affecting the individual’s sensitivity towards experiences in life after birth [5].

The importance of a prosperous prenatal environment and birth process for future health has long been recognized. Conversely, exposure to early adversity during a sensitive period of development is thought to lead to structural, physiological and metabolic changes in the fetus that increase susceptibility to later disease [6]. For instance, antenatal maternal and infant malnutrition [7, 8], antenatal maternal stress [9], low birth weight, younger maternal age and increasing birth order [10–13] have all, amongst others, been linked to poorer mental health. Particularly in autism spectrum disorder (ASD), the role of adverse prenatal and perinatal factors is well-documented. Compelling evidence exists for associations between an increased risk for ASD on the one hand and maternal infections during pregnancy, increased parental age, fetal hypoxia, prematurity, and low birth weight on the other hand [14–16]. More broadly viewed, as reported by Guedeney et al. in this issue and previously by others [17–19], associations have been found between pre- and perinatal risks and sustained social withdrawal behavior (more prevalent than ASD but considered a red flag for developing clinical disorders such as ASD, attachment disorders and depression) [20–22]. In any case, life before birth is related to mental health outcomes in life after birth.

However, it remains to be determined to what extent these factors are true risk mediators (causally involved) or rather risk indicators (linked to an increased risk but not contributing causally to the adverse outcome). Two main reasons why prenatal risks may act as risk indicators, are that these factors are associated with postnatal risks (such as social adversity and parent mental health problems) and that they relate to unmeasured confounders, such as maternally transmitted inherited factors [6]. Studies commonly deal with these two possibilities by adjusting for measured confounders. For instance, it was recently shown that when social class, family structure and religion were corrected for, prenatal circumstances were not or only marginally associated with mental health outcomes in adolescence and young adulthood [23]. Further support for the hypothesis that perinatal factors may often act as indicators instead of mediators of poor mental health was reported in a series of elegantly designed studies by Thapar and colleagues [24–26]. Using genetically informative designs, in which mothers were either genetically related or unrelated to their child as a result of assisted reproductive technologies, they showed that relationships between smoking during pregnancy and ADHD and conduct disorder symptoms, and also between breastfeeding and lower levels of conduct problems, were only present in the genetically related group and not in the genetically unrelated group [24–26]. These studies teased apart the intrauterine and genetic influences normally intertwined in explaining the fetal origins of chronic diseases and suggest pre- and perinatal risk factors may sometimes act as a proxy for the underlying genetic risk transmitted to offspring.

However, elegant as these studies may be in examining the role of prenatal risks in isolation from a genetic background, the reality of life is that the vast majority of people are conceived in the natural way and spend their life before and after birth with their biological parents. Therefore, genetic and environmental factors correlate and interact in various complex ways, causing substantial differences between people in their exposure and susceptibility to both positive and negative environmental influences [27, 28]. This differential susceptibility may be labeled as “prenatal programming of postnatal plasticity” [29] and one of the mechanisms underlying this may be epigenetics. Epigenetics can be broadly defined as those heritable changes not dependant on the genomic sequence and encompasses a variety of mechanisms to dynamically modify the expression of the genome [30]. There appear to be “discrete epigenetic checkpoints” [30] during which the genome is extra sensitive to modifications by environmental factors. This illustrates that in real life there is no effect of prenatal risk in isolation from a genetic background or vice versa.

So, in life before birth, are the dice tossed for the rest of our lives? The influence of the prenatal environment on mental health later in life is unequivocally present. Often the dice are unfairly being tossed, with some children receiving the best of odds and others the worst. However, what the best odds are is not predetermined, but may vary according to the environment in which the child grows up. In some cases, the last shall be the first.
